# Comparative Proteomic Analysis of *Gossypium thurberi* in Response to *Verticillium dahliae* Inoculation

**DOI:** 10.3390/ijms161025121

**Published:** 2015-10-22

**Authors:** Weiping Fang, Deyi Xie, Heqin Zhu, Wu Li, Zhenzhen Xu, Lirong Yang, Zhifang Li, Li Sun, Jinxia Wang, Lihong Nie, Zhongjie Tang, Shuping Lv, Fu’an Zhao, Yao Sun, Yuanming Zhao, Jianan Hou, Xiaojie Yang

**Affiliations:** 1 Economic Crop Research Institute, Henan Academy of Agricultural Sciences, Zhengzhou 450002, China; E-Mails: xiedeyi101@sina.com (D.X.); cotton169@sina.com (W.L); nlh200999@sina.com (L.N.); tangzhongjie2001@sina.com (Z.T.); lvshuping316@sina.com (S.L.); fazcotton@sina.com (F.Z.); yangming1697@sina.com (Y.S.); zzzym5@sina.com, (Y.Z.); hjnwikiscott@gmail.com (J.H.); 2 State Key Laboratory of Cotton Biology, Institute of Cotton Research, Chinese Academy of Agricultural Sciences, Anyang 455000, China; E-Mails: Heqinanyang@sina.com (H.Z.); lele20032@sina.com (Z.X.); lizhifang2015@gmail.com (Z.L.); 3 Plant Protection Research Institute, Henan Academy of Agricultural Sciences, Zhengzhou 450002, China; E-Mail: yangzi0224_sohu@sina.com; 4 Department of Biological Sciences, Texas Tech University, Lubbock, TX 79409, USA; E-Mail: li.sun@ttu.edu; 5 Department of Crop Biotechnology, Agronomy College, Henan Agricultural University, Zhengzhou 450002, China; E-Mail: wjx29287@sina.com

**Keywords:** cotton, defense response, fungus, iTRAQ

## Abstract

Verticillium wilt is threatening cotton productivity globally. This disease is caused by soil-borne *Verticillium dahliae* which directly infects cotton roots, and exclusively colonizes and occludes xylem vessels, finally resulting in necrosis, defoliation, and most severely, plant death. For the first time, iTRAQ (isobaric tags for relative and absolute quantification) was applied to screen the differentially expressed proteins of *Gossypium thurberi* inoculated with *V. dahliae*. A total of 6533 proteins were identified from the roots of *G. thurberi* after inoculation with *V. dahliae*, and 396 showed up- and 279 down-regulated in comparison to a mock-inoculated roots. Of these identified proteins, the main functional groups were those involved in cell wall organization and reinforcement, disease-resistant chemicals of secondary metabolism, phytohormone signaling, pathogenesis-related proteins, and disease-resistant proteins. Physiological and biochemical analysis showed that peroxidase activity, which promotes the biosynthesis and accumulation of lignin, was induced early in the hypocotyl after inoculation with *V. dahliae*. Similarly, salicylic acid also accumulated significantly in hypocotyl of the seedlings after inoculation. These findings provide an important knowledge of the molecular events and regulatory networks occurring during *G. thurberi*-*V. dahliae* interaction, which may provide a foundation for breeding disease-resistance in cotton.

## 1. Introduction

Cotton (*Gossypium* spp.) is the most important fiber crop and one of the sources for edible oil and protein [1]. Cotton production and yield is detrimentally affected by the soil borne pathogen *V. dahliae* [2,3,4]. Severe outbreaks of this disease can cause yield reductions of up to 30% [5]. Fungicides and chemicals means have proven ineffective at controlling Verticillium wilt, although some cultural practices such as appropriate seeding and crop rotation can suppress the development of the disease to some extent [6].

Several reports show that, although some of the main cultivars (upland cotton, *Gossypium hirsutum* L.) were sensitive to Verticillium wilt, variation in resistance to Verticillium wilt was identified among different cotton cultivars or germplasm resources [7,8]. Using these resistant cultivars or genetic resource, progress has been made to characterize cotton defense in response to *V. dahliae* infection. For example, some defense responsive genes were identified such as PR10 [9], ERF-Like transcription factor [10], anti-apoptosis [11], major latex protein [12] and a receptor-like protein [13]. In addition, cotton inoculation experiments showed that some resistance genes could be induced within 10 min in response to pathogen infection [12]. These findings indicate the complexity of cotton defense response to *V. dahliae* infection, which may involves multiple defense pathways.

In order to elucidate the molecular mechanisms leading to Verticilium wilt resistance, RNA-seq were used to analyze the differentially expressed genes of cotton in response to *V. dahliae* infection [14,15,16]. Studies have shown the involvement of some miRNAs and their respective target genes in the resistance to Verticillium wilt [17,18], while virus-induced gene silencing and proteomic studies have further characterized this response [19,20,21]. These studies indicate that genes specially involved in the metabolism of lignin, gossypol, phytohormones, and phenylalanine may play vital roles during cotton defense response to each different species of *V. dahliae*. However, the multiple molecular mechanisms are still poorly understood.

This study aimed to shed more light on the molecular mechanisms involved in the cotton defense response. We have primarily investigated the differentially expressed proteins involved in the molecular events of resistance to *V. dahliae* infection by two-dimensional electrophoresis (2-DE) and tandem time-of-flight mass spectrometry (MALDI-TOF-MS), which showed the upregulation of the proteins most likely involved in the response to biotic and abiotic stresses, signal transduction, protein processing and degradation, and other processes [8]. In this study, iTRAQ with a more sensitive and accurate protein quantification was applied to further identify differentially expressed proteins of *G. thurberi* after *V. dahliae* inoculation. Our objective was to enrich the knowledge of the molecular events involved in the cotton defense response.

## 2. Results and Discussion

### 2.1. Colonization Identification of V. dahliae in Seedling Roots after Inoculation

**Figure 1:**
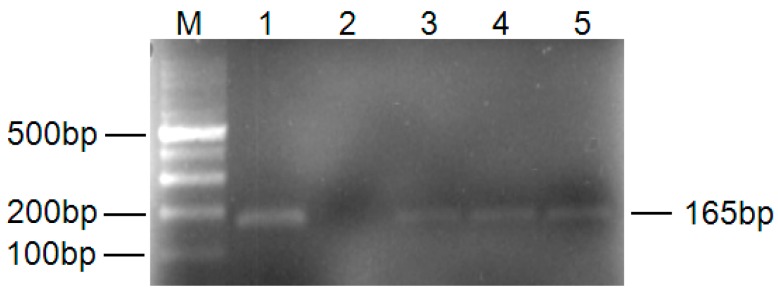
PCR amplification of tubulin β chain gene from *G. thurberi* seedlings after inoculation. **M**: Marker 100, **1**: *V. dahliae*; **2**: Control seedling; **3**–**5** represent *V. dahliae*-inoculated seedlings.

In order to identify the successful colonization of *V. dahliae* in inoculated seedlings, the pathogen- and mock-inoculated roots were sampled at 1.0 h after inoculation. The amplification of a *V. dahliae* house-keeping gene tubulin β chain using *V. dahliae-* specific primers showed a product band of 169 bp as expected (Figure 1, Table S1). The result confirmed the successful colonization of *V. dahliae* in *G. thurberi* seedlings.

### 2.2. Proteins Identified by I-TRAQ and Their Functional Annotation

**Figure 2:**
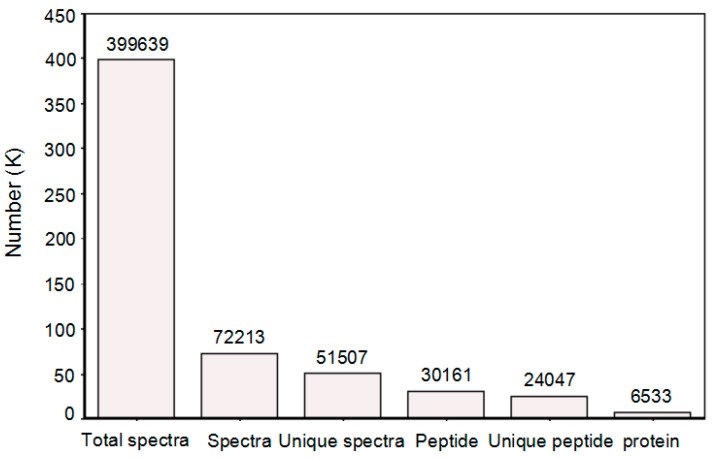
Basic statistics of identified proteins.

To obtain a relative overview of the molecular mechanism and metabolism pathways during the cotton response to *V. dahliae* inoculation, equal amounts of proteins extracted from the roots of both *V. dahliae*- and mock-inoculated seedlings at 0, 0.5, 1, 6, 12, and 24 h after inoculation were mixed thoroughly, respectively, and then used for proteomic analysis using the iTRAQ-based quantitative method and LC-ESI-MS/MS. As a result, a total of 6533 proteins were identified using the Mascot 2.3.02 search engine against a database [22] containing 40,523 protein sequences (Figure 2).

**Figure 3:**
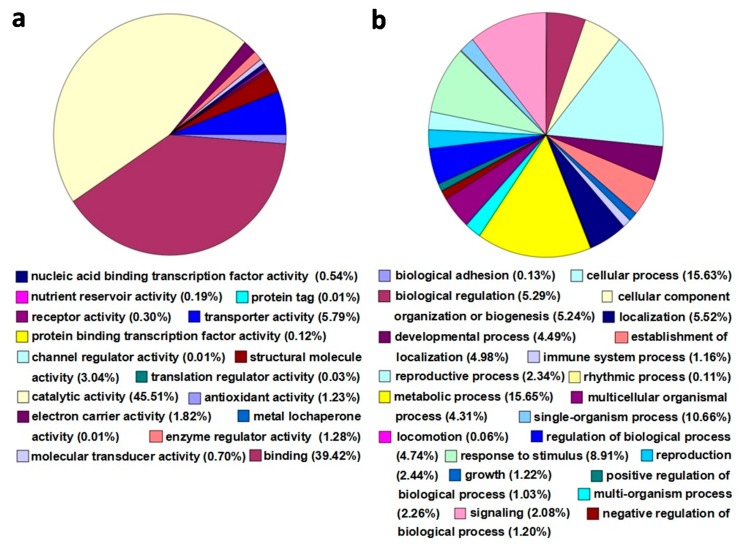
Gene Ontology (GO) annotation of the whole identified proteins. (**a**,**b**) represented the classification of molecular function and biological process of the identified proteins, respectively.

In order to investigate the functions and related biological processes of all the identified proteins, all of the total 6533 proteins were classified with Gene Ontology (GO) terms using GO::Term Finder [23]. In total, 6135 proteins were annotated, and 16 GO terms were enriched for molecular function and 22 terms for biological processing. For function classification, the 16 functional protein categories were mainly classified into catalytic activity (45.51%), followed by binding (39.42%), transporter activity (5.79%), structural molecule activity (3.04%), enzyme regulator activity (1.28%), antioxidant activity(1.23%), *etc*. (Figure 3a, Table S2). For biological processing classification, 22 categories were mainly involved in metabolic process (15.65%), followed by cellular process (15.63%), response to stimulus (8.91%), biological regulation (5.29%), *etc.* Expectedly, there were 308 proteins (1.16%) involved in the *G. thurberi* immune response to *V. dahliae* (Figure 3b, Table S2).

To further clarify the functions of the proteins in specific biological pathways, a KEGG enrichment analysis was performed. Among the total identified 6533 proteins, 4728 were assigned to 128 different specific pathways, and most of the proteins were mainly involved in metabolic pathways (1505), biosynthesis of secondary metabolites (968), ribosome (199), protein processing in the endoplasmic reticulum (172), RNA transport (152), phenylpropanoid biosynthesis (152), spliceosome (146), phytohormone signaling (132), glycolysis and gluconeogenesis (128), starch and sucrose metabolism (117). Similar to the GO analysis, 144 proteins were involved in plant-pathogen interactions (Table S3).

### 2.3. Identification and Enrichment Analysis of Differentially Expressed Proteins (DEPs)

**Figure 4:**
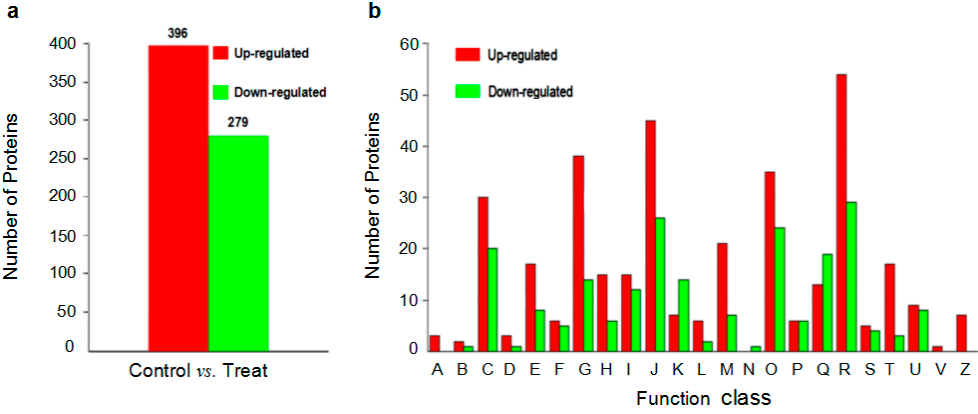
Statistics and COG (Clusters of Orthologous Groups) classification of differentially expressed proteins. (**a**,**b**) showed the statistics and the COG functional classification of differentially expressed proteins, respectively. A–V and Z represent RNA processing (A), chromatin structure and dynamics (B), energy production and conversion (C), cell cycle, cell division (D), amino acid metabolism and transport (E), nucleotide metabolism and transport (F), carbohydrate metabolism and transport (G), coenzyme metabolism and transport (H), lipid metabolism and transport (I), translation, ribosomal biogenesis and structure (J), transcription (K), DNA replication, recombination and repair (L), cell wall, membrane, and envelope biogenesis (M), cell motility (N), protein modification, turnover, and chaperones (O), inorganic metabolism and transport (P), biosynthesis, transport and catabolism of secondary metabolites (Q), general function prediction only (R), function unknown (S), signal transduction event (T), intracellular trafficking, secretion, and vesicular transport (U), defense responses (V), and cytoskeleton (Z).

In order to identify the protein expression changes in the roots between *V. dahlia*e- and mock-inoculated seedlings, a 1.2-fold cut-off (*p*-value < 0.05) was designated as the significant change standard in abundance. As a result, a total of 675 non-redundant proteins were identified, and among them, 396 showed up- and 279 down-regulation in seedlings after inoculation with *V. dahlia*e in comparison to the mock-inoculated (Figure 4a).

To further analyze the annotated DEPs related to the response of *G. thurberi* to *V. dahliae* inoculation, a COG (Clusters of Orthologous Groups) analysis of all the up- and down-regulated proteins was performed to phylogenetically analyze widespread domain families. Most of the up-regulated proteins (89.6%) proteins were grouped in 22 COG clusters. Among the COG clusters, the largest group was only general function prediction (15.2%, 54), followed by translation, ribosomal biogenesis and structure (12.7%, 45), carbohydrate metabolism and transport (10.7%, 38), protein modification, turnover, chaperones (9.9%, 35), energy production and conversion (8.5%, 30), cell wall, membrane, and envelope biogenesis (5.9%, 21), signal transduction mechanisms (4.8%, 17), *etc.* In addition, most of the proteins involved in cell wall, membrane, and envelope biogenesis, and cytoskeleton were up-regulated. Similarly, the down-regulated proteins had a similar distribution as that of up-regulated proteins except for RNA processing and modification (A), signal transduction (V), and cytoskeleton (Z) categories, which were nearly exclusively up-regulated proteins (Figure 4b, Table S4).

Furthermore, the Kyoto Encydopedia of Genes and Genomics (KEGG) enrichment analysis was conducted to filter the DEPs that corresponded to specific biological pathways. In total, 508 (75.3%) DEPs were assigned to 102 metabolic pathways. For the up-regulated proteins, 304 (75.3%) proteins were assigned to 90 pathways, which mainly included metabolic pathways (Ko01100), secondary metabolites biosynthesis (ko01110), ribosome (ko03010), the processing of protein in endoplasmic reticulum (ko04141), sucrose and starch metabolism (ko00500), *etc.* (Table 1).

Many of the up-regulated proteins were specially enriched in the pathways of pathogens response including flavonoid biosynthesis (ko00941), phenylpropanoid biosynthesis (ko00940), phenylalanine metabolism (ko00360), plant-pathogen interaction (ko04626), and phytohormone signal transduction (ko04075) (Table S2). In contrast, the down-regulated proteins associated with these aforementioned categories were much fewer, e.g., phenylpropanoid biosynthesis, plant-pathogen interaction, and phyto hormone signaling.

**Table 1:** Kyoto Encydopedia of Genes and Genomics (KEGG) pathways of Differentially Expressed Proteins (DEPs) during the response of *G. thurbri* to *V. dahliae*.

No.	Enriched KEGG Pathways	DEPs		*p*-Value
		Up (304)	Down (204)	
1	Metabolic pathways (ko01100)	104 (34.21%)	79 (38.73%)	1
2	Biosynthesis of secondary metabolites (ko01110)	69 (22.7%)	52 (25.49%)	1
3	Ribosome (ko03010)	32 (10.53%)	20 (9.8%)	1
4	Protein processing in endoplasmic reticulum (ko04141)	24 (7.89%)	2 (0.98%)	1
5	Starch and sucrose metabolism (ko00500)	21 (6.91%)	5 (2.45%)	1
6	Protein export (ko03060)	1 (0.33%)	3 (1.47%)	1
7	Phenylpropanoid biosynthesis (ko00940)	16 (5.26%)	0	1
8	Spliceosome (ko03040)	13 (4.28%)	13 (6.37%)	1
9	Flavonoid biosynthesis (ko00941)	12 (3.95%)	9 (4.41%)	1
10	Phenylalanine metabolism (ko00360)	11 (3.62%)	10 (4.9%)	1
11	Plant-pathogen interaction (ko04626)	10 (3.29%)	2 (0.98%)	1
12	Glycolysis/Gluconeogenesis (ko00010)	9 (2.96%)	4 (1.96%)	1
13	Phagosome (ko04145)	8 (2.63%)	2 (0.98%)	1
14	Endocytosis (ko04144)	7 (2.3%)	2 (0.98%)	1
15	Amino sugar and nucleotide sugar metabolism (ko00520)	7 (2.3%)	7 (3.43%)	1
16	Oxidative phosphorylation (ko00190)	7 (2.3%)	14 (6.86%)	1
17	RNA transport (ko03013)	7 (2.3%)	5 (2.45%)	1
18	Fatty acid metabolism (ko00071)	7 (2.3%)	2 (0.98%)	1
19	Ascorbate and aldarate metabolism (ko00053)	7 (2.3%)	6 (2.94%)	1
20	Aminoacyl-tRNA biosynthesis (ko00970)	7 (2.3%)	17 (8.33%)	1
21	Carbon fixation in photosynthetic organisms (ko00710)	7 (2.3%)	0	1
22	Glyoxylate and dicarboxylate metabolism (ko00630)	6 (1.97%)	0	1
23	Nitrogen metabolism (ko00910)	6 (1.97%)	3 (1.47%)	1
24	Alanine, aspartate and glutamate metabolism (ko00250)	6 (1.97%)	1 (0.49%)	1
25	Galactose metabolism (ko00052)	6 (1.97%)	4 (1.96%)	1
26	Pyruvate metabolism (ko00620)	6 (1.97%)	4 (1.96%)	1
27	Plant hormone signal transduction (ko04075)	5 (1.64%)	1 (0.49%)	1
28	Propanoate metabolism (ko00640)	5 (1.64%)	4 (1.96%)	1
29	Stilbenoid, diarylheptanoid and gingerol biosynthesis (ko00945)	5 (1.64%)	7 (3.43%)	1
30	Pyrimidine metabolism (ko00240)	5 (1.64%)	6 (2.94%)	1
31	Glycerophospholipid metabolism (ko00564)	5 (1.64%)	1 (0.49%)	1
32	Pentose phosphate pathway (ko00030)	4 (1.32%)	1 (0.49%)	1
33	Peroxisome (ko04146)	4 (1.32%)	3 (1.47%)	1
34	Limonene and pinene degradation (ko00903)	4 (1.32%)	6 (2.94%)	1
35	Terpenoid backbone biosynthesis (ko00900)	4 (1.32%)	1 (0.49%)	1
36	Tryptophan metabolism (ko00380)	4 (1.32%)	1 (0.49%)	1
37	Cutin, suberine and wax biosynthesis (ko00073)	4 (1.32%)	1 (0.49%)	1
38	Proteasome (ko03050)	4 (1.32%)	3 (1.47%)	1
39	Pentose and glucuronate interconversions (ko00040)	4 (1.32%)	4 (1.96%)	1
40	Tyrosine metabolism (ko00350)	4 (1.32%)	0	1
41	Purine metabolism (ko00230)	3 (0.99%)	8 (3.92%)	1
42	Porphyrin and chlorophyll metabolism (ko00860)	3 (0.99%)	1 (0.49%)	1
43	Glucosinolate biosynthesis (ko00966)	3 (0.99%)	0	1
44	mRNA surveillance pathway (ko03015)	3 (0.99%)	4 (1.96%)	1
45	Flavone and flavonol biosynthesis (ko00944)	3 (0.99%)	8 (3.92%)	1
46	Citrate cycle (ko00020)	3 (0.99%)	1 (0.49%)	1
47	Glycerolipid metabolism (ko00561)	3 (0.99%)	2 (0.98%)	1
48	Phosphatidylinositol signaling system (ko04070)	3 (0.99%)	0	1
49	Ubiquinone and other terpenoid-quinone biosynthesis (ko00130)	3 (0.99%)	0	1
50	Diterpenoid biosynthesis (ko00904)	3 (0.99%)	7 (3.43%)	1
51	β-Alanine metabolism (ko00410)	3 (0.99%)	3 (1.47%)	1
52	Ubiquitin mediated proteolysis (ko04120)	3 (0.99%)	0	1
53	Glycine, serine and threonine metabolism (ko00260)	3 (0.99%)	1 (0.49%)	1
54	Fatty acid biosynthesis (ko00061)	2 (0.66%)	1 (0.49%)	1
55	Lysine degradation (ko00310)	2 (0.66%)	1 (0.49%)	1
56	Cysteine and methionine metabolism (ko00270)	2 (0.66%)	2 (0.98%)	1
57	Other glycan degradation (ko00511)	2 (0.66%)	1 (0.49%)	1
58	Monoterpenoid biosynthesis (ko00902)	2 (0.66%)	0	1
59	Phenylalanine, tyrosine and tryptophan biosynthesis (ko00400)	2 (0.66%)	0	1
60	Sphingolipid metabolism (ko00600)	2 (0.66%)	2 (0.98%)	1
61	Arginine and proline metabolism (ko00330)	2 (0.66%)	1 (0.49%)	1
62	Nucleotide excision repair (ko03420)	2 (0.66%)	1 (0.49%)	1
63	Sulfur metabolism (ko00920)	2 (0.66%)	0	1
64	Glycosphingolipid biosynthesis-ganglio series (ko00604)	1 (0.33%)	1 (0.49%)	1
65	ABC transporters (ko02010)	1 (0.33%)	1 (0.49%)	1
66	α-Linolenic acid metabolism (ko00592)	1 (0.33%)	3 (1.47%)	1
67	Photosynthesis (ko00195)	1 (0.33%)	0	1
68	Tropane, piperidine and pyridine alkaloid biosynthesis (ko00960)	1 (0.33%)	2 (0.98%)	1
69	Inositol phosphate metabolism (ko00562)	1 (0.33%)	0	1
70	Anthocyanin biosynthesis (ko00942)	1 (0.33%)	0	1
71	Pantothenate and CoA biosynthesis (ko00770)	1 (0.33%)	0	1
72	Glycosaminoglycan degradation (ko00531)	1 (0.33%)	1 (0.49%)	1
73	Taurine and hypotaurine metabolism (ko00430)	1 (0.33%)	0	1
74	Histidine metabolism (ko00340)	1 (0.33%)	1 (0.49%)	1
75	Biosynthesis of unsaturated fatty acids (ko01040)	1 (0.33%)	0	1
76	Butanoate metabolism (ko00650)	1 (0.33%)	1 (0.49%)	1
77	Benzoxazinoid biosynthesis (ko00402)	1 (0.33%)	1 (0.49%)	1
78	Fatty acid elongation (ko00062)	1 (0.33%)	0	1
79	DNA replication (ko03030)	1 (0.33%)	1 (0.49%)	1
80	Isoquinoline alkaloid biosynthesis (ko00950)	1 (0.33%)	0	1
81	Glutathione metabolism (ko00480)	1 (0.33%)	7 (3.43%)	1
82	Valine, leucine and isoleucine degradation (ko00280)	1 (0.33%)	3 (1.47%)	1
83	RNA polymerase (ko03020)	1 (0.33%)	4 (1.96%)	1
84	Fructose and mannose metabolism (ko00051)	1 (0.33%)	2 (0.98%)	1
85	Cyanoamino acid metabolism (ko00460)	1 (0.33%)	0	1
86	One carbon pool by folate (ko00670)	1 (0.33%)	0	1
87	SNARE interactions in vesicular transport (ko04130)	1 (0.33%)	1 (0.49%)	1
88	Regulation of autophagy (ko04140)	1 (0.33%)	0	1
89	RNA degradation (ko03018)	1 (0.33%)	2 (0.98%)	1
90	Circadian rhythm-plant (ko04712)	1 (0.33%)	0	1
91	Zeatin biosynthesis (ko00908)	0	3 (1.47%)	1
92	Isoflavonoid biosynthesis (ko00943)	0	3 (1.47%)	1
93	Selenocompound metabolism (ko00450)	0	2 (0.98%)	1
94	Sesquiterpenoid and triterpenoid biosynthesis (ko00909)	0	1 (0.49%)	1
95	Ribosome biogenesis in eukaryotes (ko03008)	0	4 (1.96%)	1
96	Indole alkaloid biosynthesis (ko00901)	0	1 (0.49%)	1
97	Brassinosteroid biosynthesis (ko00905)	0	2 (0.98%)	1
98	Steroid biosynthesis (ko00100)	0	1 (0.49%)	1
99	Riboflavin metabolism (ko00740)	0	1 (0.49%)	1
100	Valine, leucine and isoleucine biosynthesis (ko00290)	0	1 (0.49%)	1
101	Vitamin B6 metabolism (ko00750)	0	2 (0.98%)	1
102	Base excision repair (ko03410)	0	1 (0.49%)	1

### 2.4. Proteins Related to Cell Wall Organization and Reinforcement

**Figure 5:**
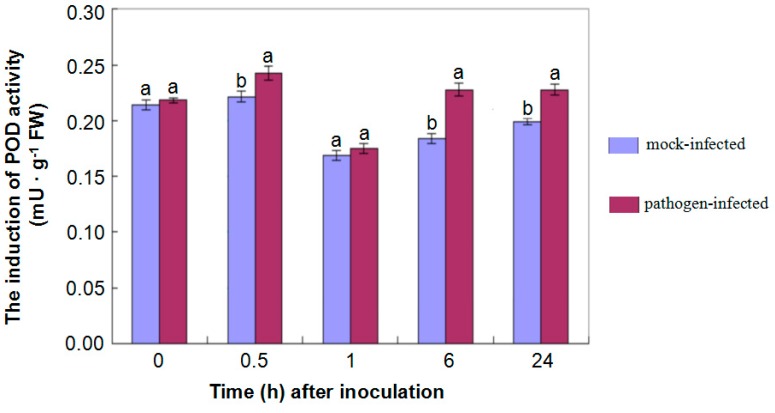
Induction of peroxidase (POD) activity in hypocotyls after inoculation. Data represented the induction of POD activity in the hypocotyls of *G. thurberi* seedlings in response to *V. dahliae* infection at 0, 0.5, 1, 6, 24 h after mock- and fungal- inoculation. Means significantly different from one another (at 0.5, 6, 24 h after inoculation) by Tukey (α = 0.05) were shown as different letters on each column.

In plants, the cell wall is one of the first defense barriers protecting plants from invasion by fungal pathogens, and is also a major factor in the basal resistance of the host plant. During pathogen attacks, the structure and composition of cell walls are modified to withstand the physical and chemical forces of fungus [24,25]. For example, some cell wall components like lignin are accumulated in the stem or roots to reinforce the cell wall against pathogen invasion [16,26,27]. In Arabidopsis inoculated with *Pseudomonas syringae* pv. tomato DC3000, the resistant mutants showed more callose deposition than wild type plants, and a susceptible mutant had reduced callose deposition [28]. Similarly, tobacoo plants also showed an increased callose deposition in leaves when attacked by *Pseudomonas syringae* [29]. In addition, significant more papillae which are effective in preventing penetration by pathogens, were found on leaves of barley during infection with *Blumeria graminis f.* sp*. Hordei* [30]. In this study, some proteins involved in lignin biosynthesis and/or metabolic processes (Cotton_D_gene_10018639; Cotton_D_gene_10007128), callose synthesis (Cotton_D_gene_10027166, Cotton_D_gene_10014770) and deposition (Cotton_D_gene_10033043, Cotton_D_gene_10014769, Cotton_D_gene_10016775) were also induced after inoculating *G. thurberi* with *V. dahliae* (Table S4). A total of 21 proteins, involved in cell wall, membrane, envelope biogenesis, and cytoskeleton, were induced in roots of *G. thurberi* after infection of *V. dahliae* in this experiment (Table S4). Previous reports have shown that, after infection of pathogens, the peroxidase (POD) activity of plants promoted the biosynthesis and accumulation of lignin, and the lignification of infected tissues [31,32]. In the present study, the measurement of POD activity also showed that the activation of POD in hypocotyl increased significantly after inoculation with *V. dahliae* compared to that of mock-inoculated control during the 24 h after treatment (Figure 5).

### 2.5. Products of Metabolism Involved in Response of G. thurberi to V. dahliae

Products of plant secondary metabolism play very important roles in plant-pathogen interaction and plant defense. In this study, proteins involved in synthesis of secondary metabolites such as phytoalexin, chorismate, and phenolics precursor flavonoids, were significantly increased in response to *V. dahliae* inoculation than that of control, which contributed to plant response to biotic stress [33]. For example, one chorismate binding protein (Cotton_D_gene_10006706), which is an important component of plant defense against pathogen attempt, was up-regulated (Table S4) [34,35]. In addition, another DEP (Cotton_D_gene_10006256) involved in phytoalexin metabolism pathway, which plays an important role during response to pathogens attack, was also significantly accumulated (Table S4) [36]. Moreover, several proteins (Cotton_D_gene_10031714, involved in phenylpropanoid biosynthetic process; Cotton_D_gene_10030618, involved in phenylpropanoid metabolic process; Cotton_D_gene_10008985, involved in phenylpropanoid metabolic process), which are the common targets of different plant pathogens, were also up-regulated (Table S4) [37,38,39,40].

### 2.6. The Differential Expression of Proteins in Signal Pathways of Phytohormones

Plant hormones play central roles in plant responses to almost all of biotic stresses, during which the plant’s own defense systems convert pathogen-induced signalling into the activation of defense responses, and most of which depend on the action of plant hormones such as salicylic acid (SA) and jasmonates (JAs), *etc.*, [41,42]. In this study, 132 proteins involved in the synthesis and signaling of phytohormones were identified, six of which were significantly differentially accumulated (Table S3 and Table 1). Studies on the biosynthesis, perception, and signaling of specific phytohormones in tobacco and Arabidopsis, proved the essential importance of phytohormones on the regulation of down-stream immune signal events. Indeed, upon pathogen attack, plants synthesize many different hormones, which lead to the activation of specific sets of defense-associated genes [42,43].

**Figure 6:**
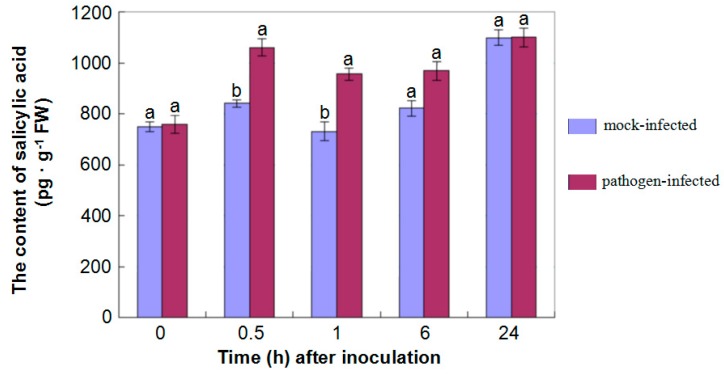
Content of salicylic acid in hypocotyls after inoculation. Data represented the content of salicylic acid in the hypocotyls of *G. thurberi* seedlings in response to *V. dahliae* infection at 0, 0.5, 1, 6, 24 h after mock- and fungal-inoculation. Means significantly different from one another (at 0.5, 1, 6 h after inoculation) by Tukey (α = 0.05) were shown as different letters on each column.

Similar results were also obtained in this study. For example, some enzymes such as isochorismate synthase (Cotton_D_gene_10000849) involved in JA biosynthesis, were up-regulated. Additionally, KEGG analysis also showed that proteins associated with several pathways involved in JA biosythesis including phenylpropanoid biosynthesis (ko00940) and phenylalanine metabolism (ko00360), displayed a significantly differential accumulation in roots after inoculation with *V. dahliae* compared to control (Table 1 and Table S4) [44]. What is more, the content quantification of SA also showed a higher abundance in hypocotyls after inoculated with *V. dahliae* compared to that of control (Figure 6).

Regulatory crosstalk is abundant in the huge, complex and still obscure network of plant hormone signaling [41,45]. Our research shows that proteins simultaneously involved in jasmonic acid, salicylic acid, abscisic acid, and ethylene signal pathways (Cotton_D_gene_10024874, Cotton_D_gene_10036611, Cotton_D_gene_10008648, Cotton_D_gene_10016775, Cotton_D_gene_10021424, Cotton_D_gene_10033223, and Cotton_D_gene_10009632) were differentially expressed, which indicates that an intricate regulatory network exists in the response of *G. thurberi* to *V. dahliae* infection (Table S4). In fact, bioinformatics analysis also showed that all of the abovementioned proteins were also involved in many other bio-processes such as regulation of protein dephosphorylation, programmed cell death, defense response through the callose deposition in cell wall, lignin biosynthetic process, oxidation-reduction process, *etc.*

### 2.7. Disease Resistance Response Proteins

**Figure 7:**
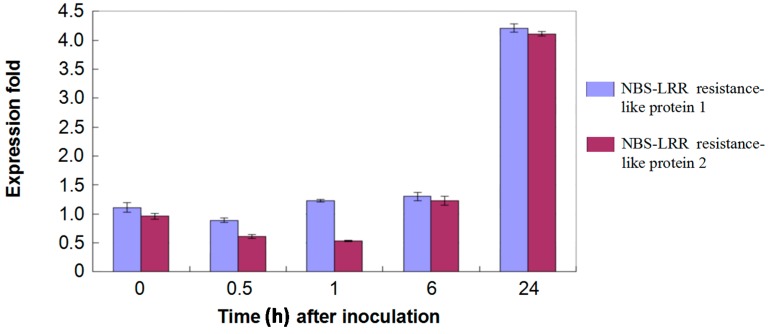
Expression pattern of 2 NBS-LRR-like protein encoding genes. Data showed the real-time RT-PCR analysis of two up-regulated protein encoding genes in response to infection at 0, 0.5, 1, 6, 24 h after mock- and fungal-inoculation.

Apart from some disease resistant proteins involved in degrading the cell wall components of pathogenic fungi such as chitinases (paralogs of Cotton_D_gene_10005890, Cotton_D_gene_10018458, Cotton_D_gene_10005890), and β-1,3-glucanase (the paralog of Cotton_D_gene_10017109), which were accumulated (Table S4) [46,47], many NBS-LRR domain containing proteins, which can recognize the effectors of pathogens through LRR domains directly or indirectly, were identified [48,49,50]. Although most of these NBS-LRR domain containing proteins showed no significant difference in cotton roots, there were two (paralogs of Cotton_D_gene_10024930 and Cotton_D_gene_10027338) which were up-regulated (Table S4). Similarly, real-time RT-PCR analysis also demonstrated that both of the two protein-encoding genes were induced at 24 h at the transcriptional level after inoculation compared to that of control (Figure 7).

## 3. Experimental Section

### 3.1. Plant Cultivation and Inoculation

A moderately aggressive defoliating fungus, *V. dahliae* D07038, from the Cotton Institute of the Chinese Academy of Agriculture Sciences (CICAAS), was incubated on a potato-dextrose agar plate for 1 week, and then inoculated for 3–4 days in Czapek broth on a shaker at 120 rpm at 25 °C until spores concentration reached 10^8^–10^9^ spores/mL. The suspension was adjusted to 10^7^ spores/mL with sterilized distilled water for inoculation [2].

Seeds of cotton (*G. thurberi*) were surface-sterilized with ddH_2_O, and planted in sterilized soil (a mix of vermiculite and nutrient) at 25 °C in a greenhouse. Every seedling was inoculated with 15 mL of a *V. dahliae* spore suspension of 10^7^ spores/mL by watering roots at the two-true-leaf developmental stage of seedlings [51]. Control seedlings were inoculated with equal volume of ddH_2_O in the same way. The roots, hypocotyls, and leaves of four individual seedlings of triple biological repeats for each treatment were harvested at 0, 0.5 1, 6, 12, and 24 h post-inoculation after washing by 75% ethanol and sterilized water [5]. They were frozen immediately in liquid nitrogen and stored at −80 °C for future use.

### 3.2. Protein Extraction and Quantification

Roots were ground into fine powder in liquid nitrogen, and then total proteins were extracted with lysis buffer (7 M Urea, 2 M Thiourea, 4% CHAPS, 40 mM Tris-HCl, pH 8.5) containing 1 mM PMSF and 2 mM EDTA (final concentration). 5 min later, 10 mM DTT (dl-Dithiothreitol) was added to each sample. The suspension was sonicated for 15 min at 200 W, and then centrifuged for 15 min at 4 °C, 30,000× *g*. The supernatant was mixed well with 5× volume of chilled acetone containing 10% (*v*/*v*) TCA (trichloroacetic acid), and then incubated overnight at −20 °C. After centrifugation of 15 min at 4 °C, 30,000× *g*, the precipitate was thoroughly washed three times with chilled acetone and air-dried, and then dissolved in lysis buffer (7 M urea, 2 M thiourea, 4% NP40, 20 mM Tris-HCl, pH 8.0–8.5). The suspension was sonicated for 15 min at 200 W and centrifuged for 15 min at 4 °C, 30,000× *g*, and the supernatant was then transferred to another new tube. In order to reduce disulfide bonds of supernatant proteins, 10 mM DTT was added and samples were incubated for 1 h at 56 °C. Subsequently, 55 mM IAM (2-iodoacetamide) was added to block the cysteines, and then incubated for 1 h in dark condition. The supernatant was mixed thoroughly with 55-fold volume of chilled acetone for 2 h at −20 °C to precipitate proteins. After centrifugation of 15 min at 4 °C, 30,000× *g*, the supernatant was discarded, the precipitate was air-dried for 5 min and dissolved in 500 µL 0.5 M TEAB (triethylammonium bicarbonate), then sonicated for 15 min at 200 W. Finally, samples were centrifuged for 15 min at 4 °C, 30,000× *g*. The supernatant was transferred to a new tube and quantified using the Bradford method using bovine serum albumin as a standard [52]. The protein solutions were kept for further analysis at −80 °C.

### 3.3. I-TRAQ Labeling and SCX Fractionation

Total proteins (100 µg) were taken out of each sample solution and digested with Trypsin Gold (Promega, Madison, WI, USA) with the ratio of protein:trypsin = 30:1 for 16 h at 37 °C. After being dried by vacuum centrifugation, the peptides were reconstituted in 0.5 M TEAB and processed according to the protocol for 8-plex iTRAQ reagent (Applied Biosystems, Waltham, MA, USA). Briefly, after one unit of iTRAQ reagent was thawed and reconstituted in 24 µL isopropanol, the peptide samples were labelled with iTRAQ tags as follow: mock-inoculated seedlings (114 tag), pathogen inoculated seedlings (118 tag). After the labelled peptides were incubated for 2 h at room temperature, the labelled peptide mixtures were then pooled and dried by vacuum centrifugation.

SCX chromatography was performed using a LC-20AB HPLC Pump system (Shimadzu, Kyoto, Japan). After reconstituted in 4 mL buffer A (25 mM NaH_2_PO_4_ in 25% ACN, pH 2.7), the labeled peptide mixtures were loaded onto a 4.6 × 250 mm Ultremex SCX column containing 5-µm particles (Phenomenex, Torrance, CA, USA). The peptides were eluted for 10 min at the flow rate of 1 mL/min with a gradient of buffer A, 5%–60% buffer B (25 mM NaH_2_PO_4_, 1 M KCl in 25% ACN, pH 2.7) for 27 min, and 60%–100% buffer B for 1 min. The system was then maintained for 1 min at 100% buffer B before equilibrating with buffer A for 10 min prior to the next injection. In order to reduce the complexity and improve the accuracy of the total labelled peptides for the following mass spectrometry analysis, the eluted peptides were collected and pooled into 20 fractions every minute by monitoring the absorbance at 214 nm, and each fractions were desalted with a Strata XC18 column (Phenomenex), and dried by vacuum centrifugation.

### 3.4. LC-ESI-MS/MS and Data Analysis

Each fraction was resuspended in buffer A (5% ACN, 0.1% FA) and centrifuged for 10 min at 20,000× *g*, the final concentration of peptide was about 0.5 μg/μL. 10 μL supernatant was loaded on a LC-20AD nanoHPLC (Shimadzu, Kyoto, Japan) by the autosampler onto the 2 cm C18 trap column. Then, the peptides were eluted onto the 10 cm analytical C18 column (inner diameter 75 μm) packed in-house. After samples were loaded for 4 min at 8 μL/min, the 35 min gradient was run at 300 nL/min starting from 2% to 35% B (95% ACN, 0.1% FA), followed by 5 min linear gradient to 60% and by 2 min linear gradient to 80%, and after maintenance for 4 min at 80% B, return to 5% within 1 min.

Data collection was performed using the TripleTOF 5600 System (AB SCIEX, Concord, ON, Canada) fitted with a Nanospray III source (AB SCIEX), and using a pulled quartz tip as the emitter (New Objectives, Woburn, MA, USA). Data was collected using an ion spray voltage at 2.5 kV, curtain gas of 30 psi, nebulizer gas of 15 psi, and an interface heater temperature at 150 °C. The MS was operated for Time of Flight Mass Spectrometry (TOF MS) scans with a reversed phase (RP) of equal to or greater than 30,000 Full Width Half Maximum (FWHM). For Information Dependent Acquisition (IDA), survey scans were acquired in 250 ms, and if exceeding a threshold of 120 counts per second (counts/s) and with a +2 to +5 charge-state, 30 product ion scans were collected. The total cycle time was set to 3.3 s, and Q2 transmission window was 100 Da for 100%. For each scan, four time bins were summed at a pulser frequency value of 11 kHz by monitoring the 40 GHz multichannel Time to Digital Convert (TDC) detector with four-anode channel detect ion. For collision-induced dissociation, the sweeping collision energy setting of 35 ± 5 eV, coupled with iTRAQ adjust rolling collision energy, was applied to all precursor ions. Dynamic exclusion was set for 1/2 of peak width (15 s), and each time, the exclusion list was refreshed off the precursor.

After collected from the Orbitrap, the raw data files were converted into Mascot generic format (MGF) files by Proteome Discoverer 1.2 (PD 1.2, Thermo), (5600 msconverter). Protein identification was performed through Mascot search engine (Matrix Science, London, UK; version 2.3.02) against a database containing 40,523 sequences [22].

For protein identification, the mass tolerance of 0.1 Da (ppm) and 0.05 Da for fragmented ions were permitted for intact peptide masses with the allowance for one missed cleavage in trypsin digests, and using Carbamidomethyl (C), iTRAQ8plex (N-term), and iTRAQ8plex (K) as fixed modifications, and Gln- > pyro-Glu (N-term Q), Oxidation (M), and Deamidated (NQ) as the potential variable modifications. At the same time, the charge states of peptides were set to + 2 and + 3. Specifically, an automatic decoy database search was performed in Mascot through choosing the decoy checkbox in which a random sequence of database is generated and tested for raw spectra and the real database. In addition, in order to further reduce the false probability in peptide identification, peptides only with the significance scores (≥20) at the 99% confidence interval by a Mascot probability analysis greater than “identity” were utilized in this study, and each confident protein identification should involve at least one unique peptide.

For protein quantitation, that a protein should contain at least two unique peptides was required. Mascot 2.3.02 was applied to weight and normalize the quantitative protein ratios with the median ratio, and the ratios only with *p*-values <0.05 and the fold changes only of >1.2 were considered as significant.

### 3.5. Bioinformatics Analysis

Cluster of Orthologous Groups of proteins (COG), a database for protein ortholog classification, was used to classify and group all the identified proteins [23].

GO enrichment analysis was applied to map all DEPs to GO terms in the database (http://www.geneontology.org/), calculating protein encoding gene numbers for every term, then using a hypergeometric test to determine significantly enriched GO terms of DEPs based on “GO::Term Finder” [53], and the algorithm method used is described as follows

(1) $$P=\sum_{i=0}^{M-1}\left\{{(}_{i}^{M}){(}_{n-i}^{N-M})\}/{(}_{n}^{N}\right)$$

where, *N* represents the number of all genes with GO annotations, *n* represents the number of DEPs in *N*, *M* represents the number of all genes that are annotated to certain GO terms, and *m* represents the number of DEPs in *M*.

KEGG, a major pathway-related database [54], was applied to perform metabolic pathway enrichment analysis of DEPs [55]. This analysis identifies significantly enriched metabolic pathways of DEPs. The calculating formula is the same as that in GO enrichment analysis, but here, *N* is the number of all proteins with known KEGG annotation, *n* is the number of DEPs in *N*, *M* is the number of all proteins annotated to specific pathways, and *m* is the number of DEPs in *M*.

### 3.6. Measurement of Enzyme Activity

The hypocotyl samples were collected at 0, 0.5, 1, 6, 12, and 24 h from both *V. dahliae*- and mock-inoculated cotton plants. Five seedlings were collected for each treatment at each time point. Samples of 100 mg were homogenized thoroughly in extraction buffer (sodium acetate 50 mM, pH 5.0). After the lysate was centrifuged for 15 min at 14,000× *g*, 4 °C, the supernatant was collected as the crude enzyme for the estimation of enzyme activities. POD activity was assayed at 470 nm by using guaiacol as the hydrogen donor [25]. The reaction mixture contained 0.1 M NaPi, 50 mM guaiacol, 10 mM H_2_O_2_, and the crude extracts. The assay was performed for 5 min at 25 °C, and enzyme activity was determined over the linear part of the reaction spectrophotometrically at 470 nm; POD activity was expressed as mU per gram fresh weight of sample.

### 3.7. Content Quantification of Hormones

The extraction, purification, and quantification of endogenous phytohormones were performed by enzyme-linked immunosorbent assay (ELISA) according to the methods with minor modification [56]. In total, 0.5 g of hypocotyl powder was homogenized by inversing in pre-chilled 80% aqueous methanol containing butoylated hydroxytoluene (1 mmol/L). The supernatant was centrifuged for 20 min at 5000× *g* at 4 °C, and the precipitate was re-extracted again. The crude extract was passed through a Sep-Pak C18 cartridge (Waters, Milford, MA, USA). Then, after the filtrate was dried in N_2_ gas, the resultant residue was dissolved in phosphate buffered saline (PBS, 0.01 mol/L, pH 7.4). The level of salicylic acid was then determined and expressed as pg per gram fresh weight of sample based on monoclonal antibodies (Affandi, Shanghai, China). Absorbance of the developed color at 490 nm was measured by a microplate reader (M-SPmax250, Wako Pure Chem, Tokyo, Japan).

### 3.8. qRT-PCR Analysis

Two induced NBS-LRR domain containing protein paralogs coding genes (Cotton_D_gene_10024930 and Cotton_D_gene_10027338) were selected for expression pattern analysis. qRT-PCR was performed to estimate the expression profile at the transcriptional level. Specific primers with 95%–103% amplification efficiency of cotton and endogenous genes were designed with Primer Premire 5.0 (Premier, Toronto, ON, Canada) (Table S1). The qRT-PCR assay using 2 μL (5 ng/μL) cDNA and SYBR Green PCR Master Mix (Takara, Dalian, China) was carried out in three technical triplicates on an ABI Prism 7000 Real-time PCR system (Foster City, CA, USA). 20 μL qRT-PCR reactions were incubated in a 96-well plate at 95 °C for 10 min, followed by 40 cycles of 95 °C for 15 s and 60 °C for 60 s with the default PCR baseline subtracted RFU of 100. The cotton endogenous cotton endogenous *actin* gene was used as the internal reference gene to normalize the amount of cDNA in each reaction (Table S1), and the relative expression levels of selected genes were calculated with the 2^−ΔΔ*C*t^ method using the SDS software (Applied Biosystems) [57].

### 3.9. Statistical Analysis

The data of phytohormone content and enzyme activity obtained in this study were subjected to analysis of variance (ANOVA), and all significant differences were examined according to Tukey test by DPS 6.05 software at *p* < 0.05 (Zhejiang University, Hangzhou, China) [58].

## 4. Conclusions

In this study, iTRAQ was applied for the first attempt to identify the differentially expressed proteins in *G. thurberi* inoculated with *V. dahliae*. As a result, 6533 proteins were identified from the roots, and among these identified proteins, 396 showed up- and 279 down-regulation after inoculation when compared to control. COG annotation showed that, 355/396 (89.6%) proteins fell into 22 COG clusters. Among these COG clusters, except for some basal metabolic groups, most of the rest, involved in cell wall, membrane, envelope biogenesis, and cytoskeleton, were up-regulated. Further KEGG analysis showed that the up-regulated proteins involved in the pathogen resistance pathway such as phenylalanine metabolism (ko00360), phenylpropanoid biosynthesis (ko00940), flavonoid biosynthesis (ko00941), plant-pathogen interaction (ko04626), and phytohormone signaling (ko04075), were enriched. Moreover, peroxidase (POD) activity, which promotes the biosynthesis and accumulation of lignin, and salicylic acid, which plays a central role in plant responses to almost all of biotic stresses, were both steadily induced in the hypocotyl of pathogen inoculated seedlings during the first 24 h after inoculation in comparison to control.

These findings provide a more comprehensive picture of the molecular events involved in regulatory networks during the response of *G. thurberi* to *V. dahliae* infection, which may support further research to breed disease-resistant cotton.

## Supplementary Materials

Supplementary materials can be found at http://www.mdpi.com/1422-0067/16/10/25121/s1.
