# Supplementary material for: Comparative Proteomic Analysis of Gossypium thurberi in Response to Verticillium dahliae Inoculation
Source: Int J Mol Sci. 2015 Oct 22;16(10):25121–40. doi: 10.3390/ijms161025121 (PMC4632794; doi:10.3390/ijms161025121)
Supplement: Supplementary file 1 [file ijms-16-25121-s001.pdf]

Supplentary Information

Table S1. The primer list of selected DEPs and endogenous gene used for qRT-PCR.

No.	Gene ID (Gene Function)	Forward Primer (5′→3′)	Reverse Primer (5′→3′)
1	Cotton_D_gene_10024930 (NBS-LRR-like protein 1)	TTTGCCTCTGGAATCATTGTAC	AAACTTCTAGCCCTTCTTTGTG
2	Cotton_D_gene_10027338 (NBS-LRR-like protein 2)	TACAAAGCGAGGACCATAAACA	TACCGTCAAGTAACAAACAAGAGG
3	AF059484 (Cotton endogenous actin)	GATTCCGGTGACGGTGTTTC	TTCATCAAGGCATCGGTTAG
4	VDAG10074 (*V. dahliae* endogenous tublin β chain)	GACTTCCGTAACGGTCGCT	TTCTTGCTCTGGACGTTGCG

Table S2. GO annotation of identified proteins.

Code	Biological Process	Number (%)	Code	Molecular Function	Number (%)
1	Biological adhesion	34 (0.13%)	1	Antioxidant activity	95 (1.23%)
2	Biological regulation	1403 (5.29%)	2	Binding	3049 (39.42%)
3	Cellular component organization or biogenesis	1391 (5.24%)	3	Catalytic activity	3520 (45.51%)
4	Cellular process	4146 (15.63%)	4	Channel regulator activity	1 (0.01%)
5	Developmental process	1191 (4.49%)	5	Electron carrier activity	141 (1.82%)
6	Establishment of localization	1321 (4.98%)	6	Enzyme regulator activity	99 (1.28%)
7	Growth	323 (1.22%)	7	Metallochaperone activity	1 (0.01%)
8	Immune system process	308 (1.16%)	8	Molecular transducer activity	54 (0.70%)
9	Localization	1384 (5.22%)	9	Nucleic acid binding transcription factor activity	42 (0.54%)
10	Locomotion	15 (0.06%)	10	Nutrient reservoir activity	15 (0.19%)
11	Metabolic process	4150 (15.65%)	11	Protein binding transcription factor activity	9 (0.12%)
12	Multi-organism process	599 (2.26%)	12	Protein tag	1 (0.01%)
13	Multicellular organismal process	1143 (4.31%)	13	Receptor activity	23 (0.30%)
14	Negative regulation of biological process	318 (1.20%)	14	Structural molecule activity	235 (3.04%)
15	Positive regulation of biological process	274 (1.03%)	15	Translation regulator activity	2 (0.03%)
16	Regulation of biological process	1257 (4.74%)	16	Transporter activity	448 (5.79%)
17	Reproduction	647 (2.44%)	-	-	-
18	Reproductive process	621 (2.34%)	-	-	-
19	Response to stimulus	2364 (8.91%)	-	-	-
20	Reproduction	647 (2.44%)	-	-	-
21	Reproductive process	621 (2.34%)	-	-	-
22	response to stimulus	2364 (8.91%)	-	-	-

Table S3. KEGG pathways of identified proteins in the response of *G. thurbri* to *V. dahliae*.

**No.**	**Pathways (ID)**	**Proteins with Annotation (4728)**
1	Metabolic pathways (ko01100)	1505 (31.83%)
2	Biosynthesis of secondary metabolites (ko01110)	968 (20.47%)
3	Ribosome (ko03010)	199 (4.21%)
4	Protein processing in endoplasmic reticulum (ko04141)	172 (3.64%)
5	RNA trsansport (ko03013)	152 (3.21%)
6	Phenylpropanoid biosynthesis (ko00940)	152 (3.21%)
7	Spliceosome (ko03040)	146 (3.09%)
8	Plant-pathogen interaction (ko04626)	144 (3.05%)
9	Plant hormone signal transduction (ko04075)	132 (2.79%)
10	Glycolysis/Gluconeogenesis (ko00010)	128 (2.71%)
11	Starch and sucrose metabolism (ko00500)	117 (2.47%)
12	Pyruvate metabolism (ko00620)	116 (2.45%)
13	Amino sugar and nucleotide sugar metabolism (ko00520)	115 (2.43%)
14	mRNA surveillance pathway (ko03015)	114 (2.41%)
15	Oxidative phosphorylation (ko00190)	106 (2.24%)
16	Purine metabolism (ko00230)	106 (2.2%)
17	Phenylalanine metabolism (ko00360)	97 (2.05%)
18	Endocytosis (ko04144)	96 (2.03%)
19	Flavonoid biosynthesis (ko00941)	96 (2.03%)
20	Phagosome (ko04145)	88 (1.86%)
21	Peroxisome (ko04146)	85 (1.8%)
22	Glutathione metabolism (ko00480)	78 (1.65%)
23	RNA degradation(ko03018)	75 (1.59%)
24	Stilbenoid, diarylheptanoid and gingerol biosynthesis (ko00945)	73 (1.54%)
25	Carbon fixation in photosynthetic organisms (ko00710)	72 (1.52%)
26	Ascorbate and aldarate metabolism (ko00053)	68 (1.44%)
27	Citrate cycle (ko00020)	67 (1.42%)
28	Pyrimidine metabolism (ko00240)	67 (1.42%)
29	Galactose metabolism (ko00052)	66 (1.4%)
30	Proteasome (ko03050)	65 (1.37%)
31	Cysteine and methionine metabolism (ko00270)	62 (1.31%)
32	Glycine, serine and threonine metabolism (ko00260)	61 (1.31%)
33	Flavone and flavonol biosynthesis (ko00944)	60 (1.27%)
34	Arginine and proline metabolism (ko00330)	58 (1.23%)
35	Ribosome biogenesis in eukaryotes (ko03008)	57 (1.21%)
36	Glyoxylate and dicarboxylate metabolism (ko00630)	55 (1.16%)
37	Ubiquitin mediated proteolysis (ko04120)	55 (1.16%)
38	Fatty acid metabolism (ko00071)	53 (1.12%)
39	Limonene and pinene degradation (ko00903)	52 (1.1%)
40	Glycerophospholipid metabolism (ko00564)	52 (1.1%)

Table S3. *Cont.*

No.	Pathways (ID)	Proteins with Annotation (4728)
41	Propanoate metabolism (ko00640)	51 (1.08%)
42	Pentose and glucuronate interconversions(ko00040)	51 (1.08%)
43	Carotenoid biosynthesis (ko00906)	51 (1.08%)
44	Zeatin biosynthesis (ko00908)	51 (1.08%)
45	Fructose and mannose metabolism (ko00051)	50 (1.06%)
46	Diterpenoid biosynthesis (ko00904)	50 (1.06%)
47	Aminoacyl-tRNA biosynthesis (ko00970)	49 (1.04%)
48	Alanine, aspartate and glutamate metabolism (ko00250)	49 (1.04%)
49	Valine, leucine and isoleucine degradation (ko00280)	47 (0.99%)
50	SNARE interactions in vesicular transport (ko04130)	46 (0.97%)
51	Nitrogen metabolism (ko00910)	45 (0.95%)
52	Pentose phosphate pathway (ko00030)	44 (0.93%)
53	Tyrosine metabolism (ko00350)	42 (0.89%)
54	Other glycan degradation (ko00511)	42 (0.89%)
55	Glycerolipid metabolism (ko00561)	42 (0.89%)
56	Fatty acid biosynthesis (ko00061)	39 (0.82%)
57	ABC transporters (ko02010)	38 (0.8%)
58	β-Alanine metabolism (ko00410)	37 (0.78%)
59	alpha-Linolenic acid metabolism (ko00592)	37 (0.78%)
60	Phenylalanine, tyrosine and tryptophan biosynthesis (ko00400)	36 (0.76%)
61	Protein export (ko03060)	35 (0.74%)
62	Tryptophan metabolism (ko00380)	35 (0.74%)
63	Terpenoid backbone biosynthesis (ko00900)	35 (0.74%)
64	Cyanoamino acid metabolism (ko00460)	35 (0.74%)
65	*N*-Glycan biosynthesis (ko00510)	34 (0.72%)
66	Nucleotide excision repair (ko03420)	34 (0.72%)
67	Porphyrin and chlorophyll metabolism(ko00860)	33 (0.7%)
68	Sulfur metabolism (ko00920)	32 (0.68%)
69	Inositol phosphate metabolism (ko00562)	32 (0.68%)
70	Sphingolipid metabolism (ko00600)	31 (0.66%)
71	Valine, leucine and isoleucine biosynthesis (ko00290)	31 (0.66%)
72	Biosynthesis of unsaturated fatty acids(ko01040)	28 (0.59%)
73	Ubiquinone and other terpenoid-quinone biosynthesis (ko00130)	28 (0.59%)
74	Butanoate metabolism (ko00650)	27 (0.57%)
75	Phosphatidylinositol signaling system (ko04070)	26 (0.55%)
76	Cutin, suberine and wax biosynthesis(ko00073)	26 (0.55%)
77	Pantothenate and CoA biosynthesis (ko00770)	26 (0.55%)
78	Brassinosteroid biosynthesis (ko00905)	25 (0.53%)
79	DNA replication (ko03030)	25 (0.53%)
80	Natural killer cell mediated cytotoxicity (ko04650)	24 (0.51%)
81	Isoflavonoid biosynthesis (ko00943)	23 (0.49%)
82	Basal transcription factors (ko04650)	23 (0.49%)

Table S3. *Cont.*

No.	Pathways (ID)	Proteins with Annotation (4728)
83	RNA polymerase (ko03020)	22 (0.47%)
84	Steroid biosynthesis (ko00100)	21 (0.44%)
85	Tropane, piperidine and pyridine alkaloid biosynthesis (ko00960)	21 (0.44%)
86	Regulation of autophagy (ko04140)	20 (0.42%)
87	Lysine degradation (ko00310)	20 (0.42%)
88	Glucosinolate biosynthesis (ko00966)	20 (0.42%)
89	Selenocompound metabolism (ko00450)	19 (0.4%)
90	Isoquinoline alkaloid biosynthesis (ko00950)	19 (0.4%)
91	Mismatch repair (ko03430)	19 (0.4%)
92	Sesquiterpenoid and triterpenoid biosynthesis (ko00909)	19 (0.4%)
93	Histidine metabolism (ko00340)	18 (0.38%)
94	Riboflavin metabolism (ko00740)	17 (0.36%)
95	Benzoxazinoid biosynthesis (ko00402)	17 (0.36%)
96	One carbon pool by folate (ko00670)	17 (0.36%)
97	Vitamin B6 metabolism (ko00750)	17 (0.36%)
98	Lysine biosynthesis (ko00300)	16 (0.34%)
99	Glycosaminoglycan degradation (ko00531)	15 (0.32%)
100	Photosynthesis (ko00195)	14 (0.3%)
101	Ether lipid metabolism (ko00565)	14 (0.3%)
102	Glycosphingolipid biosynthesis-ganglio series (ko00604)	13 (0.27%)
103	Circadian rhythm-plant (ko04712)	13 (0.27%)
104	Homologous recombination (ko03440)	13 (0.27%)
105	Fatty acid elongation (ko00062)	12 (0.25%)
106	Thiamine metabolism (ko00730)	11 (0.23%)
107	Arachidonic acid metabolism (ko00590)	10 (0.21%)
108	Base excision repair (ko03410)	9 (0.19%)
109	Glycosylphosphatidylinositol-anchor biosynthesis (ko00563)	9 (0.19%)
110	Nicotinate and nicotinamide metabolism (ko00760)	9 (0.19%)
111	Glycosphingolipid biosynthesis-globo series (ko00603)	8 (0.17%)
112	Folate biosynthesis (ko00901)	8 (0.17%)
113	Indole alkaloid biosynthesis (ko00901)	8 (0.17%)
114	Circadian rhythm - mammal (ko04710)	7 (0.15%)
115	Sulfur relay system (ko04122)	7 (0.15%)
116	Linoleic acid metabolism (ko00591)	6 (0.13%)
117	C5-Branched dibasic acid metabolism (ko00660)	6 (0.13%)
118	Taurine and hypotaurine metabolism (ko00430)	5 (0.11%)
119	Monoterpenoid biosynthesis (ko00902)	5 (0.11%)
120	Synthesis and degradation of ketone bodies (ko00072)	5 (0.11%)
121	Lipoic acid metabolism (ko00785)	3 (0.06%)
122	Photosynthesis-antenna proteins (ko00196)	3 (0.06%)
123	Caffeine metabolism (ko00232)	3 (0.06%)
124	Anthocyanin biosynthesis (ko00942)	3 (0.06%)

Table S3. *Cont.*

No.	Pathways (ID)	Proteins with Annotation (4728)
125	Other types of O-glycan biosynthesis (ko00514)	3 (0.06%)
126	Biotin metabolism (ko00780)	3 (0.06%)
127	Non-homologous end-joining (ko03450)	2 (0.04%)
128	Betalain biosynthesis (ko00965)	1 (0.02%)

Table S4. COG annotation of differentially expressed proteins in the response of *G. thurbri* to *V. dahliae*.

Code	Functional Categories	Up-Regulated		Down-Regulated	
		NO.	Proteins	NO.	Proteins
**A**	RNA processing and modification	3	Cotton_D_gene_ (10018551, 10012904, 10021434)	0	
**B**	Chromatin structure and dynamics	2	Cotton_D_gene_ (10010397, 10020008)	1	Cotton_D_gene_ (10018053)
**C**	Energy production and conversion	30	Cotton_D_gene_ (10012848, 10005039, 10019449, 10011069, 10033427, 10016342, 10007690, 10013278, 10007768, 10032002, 10022441, 10027891, 10006598, 10009473, 10039426, 10006669, 10001134, 10010340, 10013559, 10024930, 10006372, 10013854, 10033966, 10012145, 10019443, 10017916, 10006681, 10029472, 10028958, 10005804)	20	Cotton_D_gene_ (10013210, 10034808, 10034785, 10012804, 10022946, 10003001, 10012805, 10032799, 10005271, 10017991, 10024137, 10002038, 10025211^2, 10020109, 10008020, 10026306, 10015835, 10038294, 10010827)
**D**	Cell cycle control, cell division, chromosome partitioning	3	Cotton_D_gene_ (10024874, 10028350, 10036218)	1	Cotton_D_gene_ (10006863)
**E**	Amino acid transport and metabolism	17	Cotton_D_gene_ (10008366, 10038278, 10006548, 10011215, 10030583, 10030827, 10014175, 10011579, 10002405, 10037613, 10039595, 10021143, 10033386, 10010080, 10003328, 10017407, 10006706)	8	Cotton_D_gene_ (10010624, 10023991, 10005953, 10039289, 10002601^2, 10036043, 10033114)
**F**	Nucleotide transport and metabolism	6	Cotton_D_gene_ (10019245, 10019245, 10016671, 10008436, 10028401, 10003328)	5	Cotton_D_gene_ (10025910, 10021402, 10022797, 10016840, 10004476)

Table S4. *Cont.*

Code	Functional Categories	Up-Regulated		Down-Regulated	
		NO.	Proteins	NO.	Proteins
**G**	Carbohydrate transport and metabolism	38	Cotton_D_gene_ (10037302, 10028839, 10019372, 10028072, 10033809[2], 10021342, 10036658, 10018308, 10023391, 10027067, 10030859, 10001552, 10026767, 10039910, 10028824, 10031457, 10030697[2], 10002267, 10010080, 10017109, 10034052, 10012361, 10028199, 10038047, 10007128, 10018311, 10039697, 10004043, 10028958, 10015205, 10036722, 10033043, 10030188, 10022459, 10030973, 10005446)	14	Cotton_D_gene_ (10009326, 10000731, 10035883, 10009762, 10024264, 10010917, 10037042[2], 10032629, 10035029, 10032837, 10020245, 10019562, 10012877)
**H**	Coenzyme transport and metabolism	15	Cotton_D_gene_ (10035451, 10028579, 10012197, 10038278, 10025336, 10030618, 10008985, 10007695, 10010080[2], 10006256, 10006066, 10023708, 10006706[2])	6	Cotton_D_gene_ (10033904, 10000259, 10034561, 10002038, 10002601, 10012377)
**I**	Lipid transport and metabolism	15	Cotton_D_gene_ (10033223, 10031714, 10032002[4], 10039426, 10033275, 10005437, 10027411, 10022996, 10001266, 10010703, 10028954, 10021255)	12	Cotton_D_gene_ (10036532, 10025264, 10031203, 10024137, 10025265, 10026834, 10001038, 10037075, 10016847, 10015335, 10020104, 10015042)
**J**	Translation, ribosomal structure and biogenesis	45	Cotton_D_gene_ (10004494, 10027779, 10004220, 10029670, 10012775, 10011816, 10040569, 10039949[2], 10005997, 10000340, 10004694, 10006894, 10032085, 10002744, 10035490, 10010478, 10003555, 10021405, 10026406, 10013181, 10003908, 10033079, 10019660, 10022895, 10038655, 10005672, 10005537, 10009175, 10025349, 10015425[2], 10038010, 10025559, 10039147, 10012502, 10035550, 10030292, 10016717, 10027196, 10025656, 10018151, 10016925, 10006046)	26	Cotton_D_gene_ (10020726, 10019584, 10022886, 10015027, 10020493, 10000394, 10035254, 10006931, 10028826, 10022637, 10017767, 10021366, 10023027, 10022284, 10009428, 10032067, 10019410, 10010148, 10020492, 10001782, 10033867, 10021472, 10030291, 10018270, 10027511, 10020964)
**K**	Transcription	7	Cotton_D_gene_ (10024874, 10020211, 10003392, 10033732, 10004346, 10006723, 10010164)	14	Cotton_D_gene_ (10031155, 10000861, 10011464, 10001782, 10003287, 10000620, 10035231, 10021472, 10007924[2], 10021957, 10010182, 10012031, 10008746)

Table S4. *Cont.*

Code	Functional Categories	Up-Regulated		Down-Regulated	
		NO.	Proteins	NO.	Proteins
**L**	Replication, recombination and repair	6	Cotton_D_gene_ (10024874, 10033732, 10004346, 10006723, 10029307, 10010164)	2	Cotton_D_gene_ (10001782, 10021472)
**M**	Cell wall/membrane/ envelope biogenesis	21	Cotton_D_gene_ (10037302, 10026791, 10039005, 10022541, 10011093, 10033809[2], 10019860, 10036658, 10039012, 10015145, 10030618, 10008985, 10015271, 10002267, 10003140, 10034052, 10034771, 10007128, 10016566, 10033043)	7	Cotton_D_gene_ (10000731, 10035883, 10000259, 10037042[2], 10013325, 10008902)
**N**	Cell motility	0		1	Cotton_D_gene_ (10034230)
**O**	Posttranslational modification, protein turnover, chaperones	35	Cotton_D_gene_ (10032487, 10010440, 10032208, 10002753, 10025340, 10012471, 10036137, 10011573, 10025013, 10038223, 10018001[2], 10002643, 10033427, 10016342, 10010034, 10007690, 10039030, 10003365, 10006598, 10006598, 10004913, 10037266, 10022952, 10023691, 10029813[2], 10032203, 10010086, 10017916, 10032501[2], 10021434, 10025870)	24	Cotton_D_gene_ (10017225, 10008783, 10017886, 10039046, 10038649, 10019135, 10011190, 10011199, 10028684, 10011182, 10011183, 10011191, 10013208, 10023402, 10006562, 10035894, 10031521, 10021857, 10013391, 10016982, 10001782, 10038294, 10026358, 10010827)
**P**	Inorganic ion transport and metabolism	6	Cotton_D_gene_ (10039610, 10000469, 10026942, 10022908, 10008648, 10003600)	6	Cotton_D_gene_ (10006229, 10020245[2], 10034136, 10036058, 10003071)
**Q**	Secondary metabolites biosynthesis, transport and catabolism	13	Cotton_D_gene_ (10018639, 10029746, 10031714, 10014089, 10023571, 10000279, 10030618, 10008985, 10029748, 10028954, 10006681, 10006706, 10021255)	19	Cotton_D_gene_ (10034112, 10005900, 10006923, 10038440, 10033018, 10000259, 10025264, 10031203, 10025265, 10031935, 10011253, 10027616, 10017861, 10036997, 10000588, 10028992, 10026694, 10020104, 10018053)

Table S4. *Cont.*

Code	Functional Categories	Up-Regulated		Down-Regulated	
		NO.	Proteins	NO.	Proteins
**R**	General function prediction only	54	Cotton_D_gene_ (10005039, 10040312, 10038273, 10005890, 10034401, 10015444, 10029450, 10016775, 10018458, 10014089, 10034022, 10027891 [2], 10009473, 10019851, 10016445, 10018137, 10024874, 10024874, 10028350, 10020278, 10030618, 10017137, 10008985, 10004109, 10014921, 10009563, 10010080, 10000976, 10033732, 10004346, 10034052, 10015382, 10035884, 10018331, 10011559, 10029623, 10004024, 10019443, 10006723, 10006681 [2], 10004043, 10017407, 10006066, 10016537, 10001937, 10010164, 10003972, 10015985, 10021434, 10004308, 10003385, 10021762)	29	Cotton_D_gene_ (10013210, 10025048, 10021040, 10022821, 10038440, 10022946, 10032264, 10000259, 10025264, 10031699, 10031203, 10040650, 10025265, 10025211, 10029866, 10016669, 10034350, 10030133, 10039970, 10003042, 10010876, 10036958, 10025176, 10035783, 10011552, 10004548, 10028992, 10022060, 10032980)
**S**	Function unknown	5	Cotton_D_gene_ (10020440, 10022964, 10031677, 10002127, 10033570)	4	Cotton_D_gene_ (10004276, 10024291, 10015806, 10009346)
**T**	Signal transduction mechanisms	17	Cotton_D_gene_ (10035405, 10011661, 10011672, 10022271 [3], 10024874, 10024874, 10028350, 10005348, 10033732, 10004346, 10012904, 10006723, 10011886, 10010164, 10013517)	3	Cotton_D_gene_ (10040577, 10027129, 10000390)
**U**	Intracellular trafficking, secretion, and vesicular transport	9	Cotton_D_gene_ (10029348, 10018287, 10007441, 10016587, 10040618, 10024449, 10036216, 10021434, 10009632)	8	Cotton_D_gene_ (10039289, 10031886, 10006309, 10013391, 10010757, 10016982, 10015777, 10016847)
**V**	Defense mechanisms	1	Cotton_D_gene_ (10033155)	0	
**Z**	Cytoskeleton	7	Cotton_D_gene_ (10032154, 10003749, 10021918, 10003751, 10024874, 10028350, 10036218)	0	
